# Baseline gene expression and dynamic endocrine therapy use in ER+ breast cancer: associations with recurrence in a real-world cohort

**DOI:** 10.21203/rs.3.rs-9998817/v1

**Published:** 2026-06-30

**Authors:** Veronica Jones, Yongzhe Wang, Christine Quinones, Dana Aljaber, Eva Nelson, Aritro Nath, Irene Kang, Hope Rugo, Lisa Yee, Victoria Seewaldt, Joanne Mortimer

**Affiliations:** City Of Hope National Medical Center; City of Hope; City Of Hope National Medical Center; University of California, Davis; University of California, Riverside; City of Hope National Medical Center; City of Hope National Medical Canter; City of Hope National Medical Canter; City of Hope Comprehensive Cancer Center; City of Hope National Medical Canter; City of Hope

**Keywords:** Endocrine therapy, Estrogen Receptor-positive breast cancer, gene expression, recurrence, side effect, endocrine therapy switching

## Abstract

Endocrine therapy (ET) selection in estrogen receptor–positive breast cancer (ER + BC) is guided by menopausal status and tolerability rather than tumor biology, despite substantial heterogeneity in recurrence. We hypothesized that baseline gene expression differentially associates with recurrence depending on initial ET class. In a retrospective cohort of 74 ER+/HER2– patients treated with adjuvant ET, we profiled baseline tumor RNA and fitted adjusted Cox models for gene and ET interactions on time-to-recurrence and time-to-switch. Among selective estrogen receptor modulators-initiated patients, higher expression of *TP53*, *WNT7B*, *UBE2T*, *BAG1*, and *ACTR3B* was associated with markedly increased recurrence, while no comparable association was observed among aromatase inhibitors-initiated patients. Forty-one percent of patients switched ET at least once, predominantly due to intolerance (joint pain, unspecified side effects); switching was not consistently associated with tumor biology. These hypothesis-generating findings identify candidate gene and ET interactions and motivate validation in larger, independent cohorts.

## 1. Introduction

Breast cancer (BC) remains the most frequently diagnosed cancer in women and the second leading cause of cancer-related mortality [1]. Estrogen receptor–positive (ER+) disease accounts for approximately 70% of new diagnoses [2], and contributes the greatest absolute number of BC-related deaths despite favorable short-term survival [3]. Unlike ER-negative tumors, which recur predominantly within five years, ER+BC exhibits a persistent recurrence risk extending more than two decades [4–6]. Endocrine therapy (ET) is the cornerstone of adjuvant treatment and significantly reduces recurrence, yet up to 30% of ER+ tumors display inherent or acquired resistance [7].

Current immunohistochemistry-based classification captures receptor presence rather than functional sensitivity, leaving marked biological heterogeneity within the ER+ subtype unresolved. Molecular assays such as Oncotype DX and MammaPrint have transformed chemotherapy decision-making [8–10], and the Breast Cancer Index informs ET duration [11], but no biomarker guides whether ET should be initiated or which agent best matches tumor biology. Evidence indicates that circumvention of estrogen signaling and proliferative activity underlie ET resistance, and that baseline gene signatures may inform recurrence risk [12–14].

ET is also frequently complicated by toxicities—vasomotor symptoms, bone loss, and a broad spectrum of intolerance effects [15–22]—that compromise adherence and drive discontinuation or agent switching [16–18,21,22]. Because discontinuation worsens recurrence-free survival (RFS), guideline-concordant management of side effects through agent switching has been advocated [22]. However, few studies have characterized ET switching patterns or their accompanying symptoms in routine practice [19], and real-world cohorts show lower adherence than clinical trials. The complex interplay among ET agents, side effects, and clinical and biological factors remains poorly understood. Notably, despite validated gene expression assays for prognostication and chemotherapy-benefit prediction, no molecular biomarker currently guides the choice between aromatase inhibitors (AIs) and selective estrogen receptor modulators (SERMs) as initial ET—a decision driven almost entirely by menopausal status and tolerability rather than tumor biology.

In the era of precision medicine, there is a need to identify as early as possible which ET agent will minimize risk of ER+BC recurrence. Tumor biology can be used to stratify efficacy of ET agent and this should be incorporated into decision-making for ET. Herein, we hypothesized that baseline tumor gene expression differentially associates with recurrence depending on the class of ET initiated. In parallel, we examined associations between baseline gene expression and ET switching, together with self-reported intolerance symptoms at switching, to explore biological factors that may contribute to treatment tolerability. By integrating recurrence outcomes, ET exposure patterns, switching behavior, and patient-reported symptoms in a real-world cohort with long-term follow-up, our study offers insight into the interplay between tumor biology and ET experience and aims to motivate more individualized ET management in ER+BC.

## 2. Methods

### 2.1 Study population

We retrospectively identified HER2–, ER + BC specimens resected at a national cancer center (2012–2016) from surgical pathology archives. Eligible patients had tumors ≥ 2 cm or <2 cm with biopsy-proven nodal metastasis, ≥ 10% ER positivity, at least 3 years of follow-up, and no metastatic disease at time of initial diagnosis. All patients received adjuvant ET, including SERMs, selective estrogen receptor degraders (SERDs), or AIs. Recurrence, defined as locoregional or distant disease ≥ 3 months post-surgery, was ascertained from clinical records. Collected data included patient-related factors (race/ethnicity, menstrual status at diagnosis, age at diagnosis, body mass index [BMI] at diagnosis, diabetes status at diagnosis), tumor-related variables (histology, grade, clinical and pathologic stage). The primary outcome was BC recurrence. Tumors were analyzed using a custom NanoString nCounter panel of 145 genes (128 endogenous, 17 housekeeping), incorporating PAM50 genes, across key pathways (ER, proliferation, EMT, PI3K, immune) as detailed in earlier reports [23]. RNA was extracted from FFPE blocks, quality-checked by spectrophotometry and bioanalyzer, hybridized with codesets, and quantified on the nCounter platform. Patients provided written informed consent (IRB 07047), and study procedures were approved under IRB 18423 in accordance with the Declaration of Helsinki.

### 2.2 Statistical analysis

We analyzed patients treated with adjuvant ET after definitive BC surgery. Patients were categorized by their initial ET type: AI-initiated if the first ET agent was an AI (anastrozole, letrozole, or exemestane), or SERM-initiated if the first agent was a SERM (tamoxifen or toremifene). We compared groups using Fisher’s exact tests for categorical variables and Kruskal–Wallis tests for continuous variables.

We considered two time-to-event outcomes. Time-to-recurrence was defined as the time from definitive surgery to BC recurrence. Time-to-switch was defined as the time from initiation of ET to each subsequent change in ET class (AI or SERM). Patients could undergo multiple switching events, and data were structured to accommodate recurrent events and time-varying covariates. In secondary description, we depicted patient flow from initial ET through switching to recurrence using an alluvial plot and summarized the distribution of self-reported symptoms among those who switched ET. We also reported the ET class exposure pattern (AI-only, SERM-only, or AI+SERM) among switchers.

We first evaluated the unadjusted association of ET type with recurrence using the Kaplan–Meier estimator and log-rank test. To explore unadjusted relationships for both outcomes, we fit bivariate Cox proportional hazards models for each baseline characteristic (age, stage, etc.) separately. These models were used for descriptive purposes only and did not guide covariate selection in multivariable analyses.

For the primary analysis of time-to-recurrence, we fit multivariable Cox models for each gene. Each model included: the continuous standardized gene expression (per 1-SD increase), initial ET type (AI vs SERM), and their interaction. These interaction terms test whether gene associations with recurrence differ by initial ET. To focus interpretation on our main objective, we highlighted genes with significant interactions (false discovery rate [FDR] < 0.05 by Benjamini–Hochberg). We visualized the hazard ratios (HRs) and 95% confidence intervals for these genes (and ET main effects) in a forest plot.

For time-to-switch, we also fit Cox models for each gene. First, we modeled time-to-switch with gene, initial ET type, and their interaction, analogous to above. Second, we modeled time-to-switch with gene, current ET type (time-varying: AI vs SERM), and their interaction. These models assess gene-dependent differences in switching intensity across initial and current ET strategies, respectively. We similarly summarized significant interactions with forest plots.

All Cox models were adjusted for baseline prognostic factors: age at diagnosis, BMI, menopausal status at diagnosis, stage, tumor grade, and histologic subtype. Proportional hazard assumption was validated by Schoenfeld residuals tests. These covariates were selected a priori as key clinical confounders of the ET–recurrence relationship. Meanwhile, to assess robustness, we performed sensitivity analyses consisting of unadjusted models and a postmenopausal-only analysis for time-to-recurrence outcome. Two-sided P values < 0.05 were considered statistically significant. All analyses were performed in R 4.1.1.

## 3. Results

### 3.1 Demographic and clinical characteristics of the cohort

**Figure 1:**
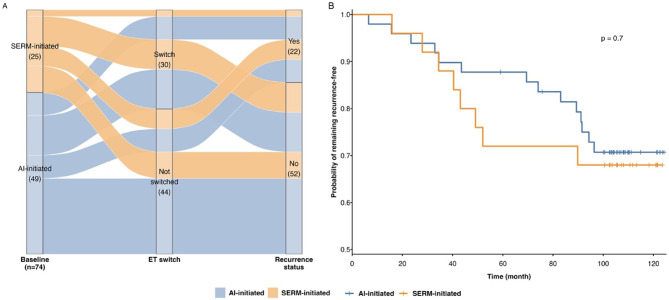
Endocrine therapy initiation and switching trajectories in relation to recurrence outcomes (A) Sankey diagram illustrating patient trajectories from initial endocrine therapy (ET) initiation (aromatase inhibitor [AI]-initiated vs selective estrogen receptor modulator [SERM]-initiated) to subsequent switching status (switched vs not switched) and recurrence outcome (yes vs no). Flow widths are proportional to the number of patients in each pathway, summarizing transitions across the treatment course (AI-initiated, blue; SERM-initiated, orange). (B) Kaplan–Meier curves for recurrence-free survival stratified by initial endocrine therapy (AI-initiated, blue; SERM-initiated, orange). Initial ET was defined as the first recorded endocrine therapy during follow-up. Survival distributions were compared using the log-rank test (p = 0.70).

The cohort included ER + BC patients treated with two initial ET patterns: AI-initiated (n = 49) and SERM-initiated (n = 25). The majority of patients (66%) initiated AI therapy, and 41% were treated with at least two ET agents during follow-up, reflecting heterogeneity in real-world ET use (Table 1). Most patients were non-Hispanic White (54%), postmenopausal (64%), and had grade II tumors (80%) and stage I/II disease (73%). Overall, 30% of patients experienced disease recurrence during follow-up. Most patients (65%) received ET for more than five years. Across individual agents, letrozole was the most commonly used (47%), followed by tamoxifen (46%) and anastrozole (39%), while exemestane (15%), fulvestrant (1%), and toremifene (1%) were less frequently prescribed. Treatment initiation and switching patterns in relation to recurrence outcomes are illustrated in Fig. 1A. Among 74 patients, 30 experienced at least one ET switch. Twenty-two patients developed recurrence. Recurrence-free survival did not differ significantly between initial ET types in unadjusted Kaplan–Meier analyses (p = 0.70; Fig. 1B). Although menstrual status and duration of ET differed significantly between groups, other baseline characteristics were generally similar across initial ET types.

### 3.2 Exploratory time-to-recurrence analysis

**Figure 2:**
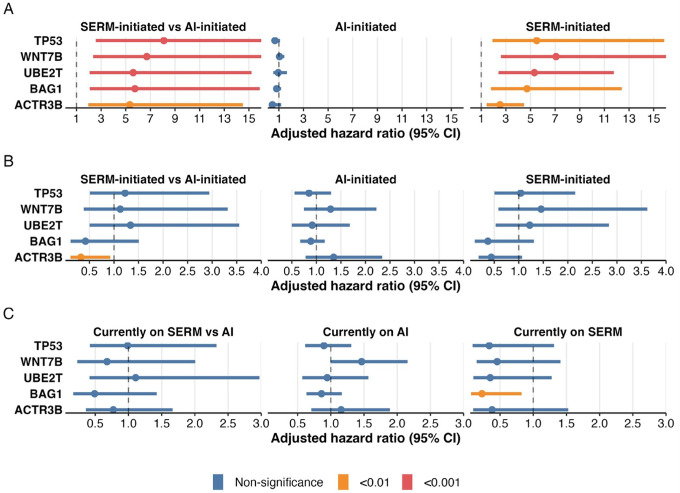
Gene expression–endocrine therapy main and interaction effects on recurrence and treatment switching outcomes Cox proportional hazards models estimating adjusted hazard ratios and 95% confidence intervals (CIs) for both main and interaction effects of continuous gene expression and initial/current endocrine therapy type. (A) Time to recurrence: interaction effects comparing selective estrogen receptor modulator (SERM)-initiated vs aromatase inhibitor (AI)-initiated therapy, along with stratum-specific main effects within AI-initiated and SERM-initiated groups. (B) Time to endocrine therapy switching: interaction effects between gene expression and initial ET type, with corresponding stratum-specific main effects. (C) Time to endocrine therapy switching: interaction effects between gene expression and current ET type, with corresponding stratum-specific main effects within each treatment group. All models were adjusted for age at diagnosis, body mass index, diabetes status at diagnosis, menopausal status at diagnosis, cancer stage, tumor size, tumor grade, and histologic subtype. Displayed genes were pre-selected based on statistically significant interaction effects in panel (A) after multiple testing correction using the Benjamini–Hochberg method, and further prioritized based on the magnitude of interaction hazard ratios. Point estimates are color-coded by statistical significance: non-significant (blue), p<0.01 (orange), and p<0.001 (red).

AI-initiated was used as the reference group across all models. Overall, baseline gene expression levels demonstrated heterogeneous associations with recurrence across initial ET strata, suggesting potential gene–treatment interaction patterns in this real-world cohort. Although gene expression profiles were not available at the time of ET assignment, we observed differences in RNA expression distributions across groups (Fig. 2A).

We identified that high expression of *TP53*, *WNT7B*, *UBE2T*, *BAG1*, and *ACTR3B* was consistently associated with markedly increased recurrence risk among SERM-initiated patients (HR range: 2.53–7.07; all adjusted p < 0.05), whereas no clear association was observed among AI-initiated patients (Table S1). The significant interaction terms for these genes further indicate that the prognostic association of gene expression differed by initial ET type, with a stronger adverse association observed in the SERM-initiated group (HR range: 5.33–8.09; all adjusted p < 0.05).

Beyond these five genes, several additional genes demonstrated similar directional patterns at the nominal level, although these associations did not persist after multiple-testing correction. Specifically, high expression of *PIK3R5*, *IGF1*, and *ITPR1* was more strongly associated with lower recurrence risk among SERM-initiated patients compared with AI-initiated patients (HR range: 0.20–0.31; all p < 0.05). In contrast, high expression of *MELK*, *CCND1*, *MMP11*, *MAPT*, *KRT17*, *FOXA*, *BRCA1*, *CDC6*, *UBE2C*, *MKI67*, *CDCA1*, *MYBL2*, *PSPHL*, *TP63*, and *ANLN*, was associated with substantially increased recurrence risk, with stronger effects observed among SERM-initiated patients than AI-initiated patients (HR range: 2.15–76.89; all p < 0.05).

Collectively, these findings suggest that, for a subset of baseline genes, high expression may be associated with greater recurrence vulnerability specifically within the SERM-initiated group, whereas the same genes show limited or no corresponding association among AI-initiated patients. In contrast to the heterogeneous gene-level findings, baseline clinical characteristics were not strongly associated with time-to-recurrence in this cohort, independent of gene expression (Table S2).

Regarding the sensitivity analyses, unadjusted models yielded interaction patterns and nominal significance concordant with the fully adjusted models, indicating the signals were not artifacts of covariate specification (Table S3). In the postmenopausal-only analysis, *WNT7B* and *TP53* retained similar interaction patterns and nominal significance, while *UBE2T*, *BAG1*, and *ACTR3B* did not, thus reflecting both potential residual confounding and limited power (Table S4).

### 3.3 Endocrine therapy switching analysis

**Figure 3:**
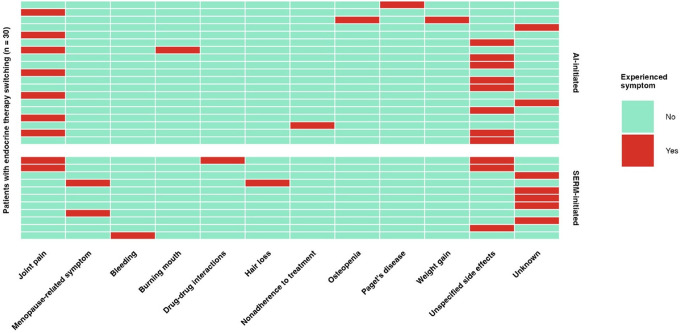
Patient-by-symptom heatmap of self-reported reasons for endocrine therapy switching, stratified by initial endocrine therapy types Each row represents an individual patient who switched at least once endocrine therapy agent, and each column denotes a self-reported symptom prompting treatment modification. Patients are grouped by initial endocrine therapy type (aromatase inhibitor [AI]-initiated and selective estrogen receptor modulator [SERM]-initiated). Red indicates the presence of a symptom and green indicates absence; patients may report multiple symptoms.

Given the heterogeneity of ET use in this real-world cohort, switching between endocrine agents was common. Because ET switching frequently occurs in response to treatment intolerance, symptom reports for patients with ET switching were visualized (Fig. 3). Among patients receiving ET regimens starting with AI, most (17/19) reported at least one symptom at any time of ET switching. Comparatively, among patients initializing ET with SERM, six (6/11) patients reported at least one symptom when ET switching occurred. Specifically, joint pain was the most frequently reported intolerance symptom, occurring in seven and two patients in AI-initiated and SERM + AI groups, respectively.

Overall, symptom reporting was heterogeneous, with most patients experiencing few symptoms across categories. Joint pain and unspecified side effects were among the more commonly reported symptoms, whereas other adverse events such as bleeding, burning mouth, and drug–drug interactions were infrequent. No consistent clustering of symptom patterns was observed, suggesting that ET switching was driven by diverse and individualized symptom experiences rather than a single dominant toxicity profile.

Looking at the aspect of ET class exposure pattern (Table S5), the majority were exposed to both AI and SERM agents (17), followed by AI-only (11) and SERM-only (2). Overall, symptom reporting was heterogeneous, with unspecified side effects (37%) and joint pain (30%) being the most frequently reported. Joint pain was more common among AI-only patients (45%), whereas unspecified side effects were most prevalent in the AI+SERM group (47%). Other symptoms were infrequent and sparsely distributed across groups. No statistically significant differences in symptom distribution were observed across ET exposure patterns. In terms of ET agent use, letrozole was the most commonly prescribed AI, particularly among AI+SERM patients. Treatment duration was similar across groups (p = 0.80).

In addition to gene-level analyses, histology and tumor grade were associated with time-to-switch outcomes (Table S2). Compared with invasive ductal carcinoma (IDC), non-ductal histology was associated with a higher likelihood of endocrine therapy switching, whereas patients with grade II tumors were less likely to switch compared with those with grade I tumors.

## 4. Discussion

In this real-world cohort of ER + BC patients treated with adjuvant ET, baseline gene expression was associated with heterogeneous recurrence risk across initial ET strata, reflecting the biological diversity of endocrine-responsive disease. Rather than implying deterministic treatment effects, these findings provide a hypothesis-generating framework in which tumor-intrinsic molecular features may interact with ET strategy to shape recurrence outcomes in routine practice.

As demonstrated in clinical trials, AI has increased efficacy with better control and increased recurrence-free survival among select populations. For example, in the Arimidex, Tamoxifen, Alone or in Combination trial, compared to tamoxifen, anastrozole was associated with prolonged disease-free survival and time-to-recurrence as well as reduced distant metastases among postmenopausal women [24]. However, in other trials such as the Tamoxifen Exemestane Adjuvant Multinational trial, no significant difference was demonstrated in disease-free survival between exemestane alone or in combination with tamoxifen [12]. Conversely, in the Suppression of Ovarian Function Trial and the Tamoxifen and Exemestane Trial, recurrence-free survival was improved in pre-menopausal patients exposed to exemestane and ovarian suppression versus tamoxifen with ovarian suppression. While pivotal in guiding our choice of endocrine therapy based on menopausal status, these studies do not offer insight regarding choice of AI, nor do they leverage genomic expression to guide addition of therapeutic options. Currently, addition of therapeutic agents, such as CDK 4/6 inhibitors, is guided by tumor size, grade, and nodal status, and molecular assays predictive of chemotherapy benefit only (e.g. Oncotype) [25]. In the era of precision medicine, there is an urgent need to tailor use of ET based on predicted efficacy. This study highlights that there may be underlying biologic mechanisms driving this differential response to therapy.

We identified that among SERM-initiated patients, high expression of *TP53*, *WNT7B*, *UBE2T*, *BAG1*, and *ACTR3B* was associated with markedly worse recurrence, whereas these associations were attenuated or absent among AI-initiated patients. These five genes converge on biological processes central to endocrine resistance: cell-cycle regulation (*TP53*), WNT pathway activation (*WNT7B*), ubiquitination and protein turnover (*UBE2T*), anti-apoptotic signaling (*BAG1*), and cytoskeletal remodeling (*ACTR3B*) [26–34]. *TP53* alterations are established contributors to endocrine resistance in ER + BC [27], although prior studies have not distinguished differential sensitivity across ET classes; our findings suggest *TP53*-related resistance may be more pronounced under SERM-based therapy. *WNT7B* activation has been linked to SERM resistance [35], and E2 ubiquitin-conjugating enzymes regulate *β*-catenin stability and WNT pathway activation [36], providing a coordinated mechanism in which altered ubiquitin-mediated protein turnover stabilizes proliferative and adaptive signaling to facilitate endocrine escape. *ACTR3B*, though not a canonical endocrine resistance gene, encodes an actin cytoskeletal component [37]; its association may reflect broader adaptive resistance processes beyond classical signaling pathways. The stronger associations observed among SERM-initiated patients suggest that tumors with elevated expression of these genes may be less effectively suppressed by receptor modulation alone than by estrogen deprivation.

*BAG1* warrants separate comment. In contrast to prior reports associating higher *BAG1* expression with favorable prognosis in ER + BC [38], we observed worse recurrence outcomes among SERM-initiated patients with elevated *BAG1*. This apparent discrepancy aligns with evidence that *BAG1* overexpression can promote SERM resistance [33, 38], emphasizing its context-dependent role in endocrine response.

Beyond recurrence, our study examined ET switching as a manifestation of real-world treatment heterogeneity. Among patients who switched and reported side effects, 63% (19/30) initiated therapy with AIs and five switched more than twice, reflecting complex longitudinal treatment trajectories (Table S5). Pre-recurrence switching was most often accompanied by patient-reported intolerance symptoms, emphasizing switching as a clinically meaningful phenotype of treatment tolerability. Prior studies have reported inconsistent switching patterns—some predominantly SERM-to-AI driven by side effects [16], others more frequent symptoms with SERMs [20]—highlighting ongoing complexity in ET management. Consistent with these reports, joint pain was the most frequently reported intolerance symptom in our cohort [15, 16, 20], and commonly accompanied switching (Fig. 3).

Few studies have directly examined biological correlates of ET switching or intolerance. Most prior work has addressed this indirectly through aromatase inhibitor–induced musculoskeletal symptoms (AIMSS) and toxicity-driven discontinuation, linking AIMSS to estrogen-responsive regulation of *TCL1A* and inflammatory pathways [39–41] and identifying genetic variants associated with AIMSS and premature AI discontinuation [42–44]. Observational studies show that toxicity-related discontinuation is common and that subsequent switching often improves tolerability [42, 45], a strategy supported by contemporary ASCO guidance [43]. However, these studies generally consider symptoms or discontinuation in isolation without integrating molecular features with longitudinal ET trajectories. Our analysis links baseline molecular characteristics with real-world switching patterns, though gene-level associations with switching were modest in effect size (Tables S6, S7) and are presented descriptively to identify signals for further investigation rather than to imply clinical discrimination.

Our findings align with a growing body of literature developing gene expression–based biomarkers to refine ET strategies in ER + BC, including signatures capturing endocrine resistance phenotypes, early genomic alterations following estrogen suppression [46], prognostic models such as ENDORSE [47], and genomic assays informing ET duration, escalation, or de-escalation [48]. However, most prior biomarker work models outcomes under a single agent or a limited set of predefined trajectories [46, 47], whereas our real-world data show that patients frequently switch between agents, largely driven by tolerability rather than disease progression. This highlights a gap in the current precision ET literature: existing signatures rarely incorporate the dynamic nature of ET delivery or the impact of side effects on adherence and treatment continuity [47–49]. Signatures developed under static treatment assumptions may have limited ability to capture the complexity of real-world ET exposure.

These results motivate a broader framework for future gene signature development. Beyond predicting antitumor efficacy [46–50], next-generation signatures may need to integrate biological determinants of treatment benefit with susceptibility to adverse effects and patterns of treatment adherence, balancing efficacy with quality of life. Such an approach could better reflect the longitudinal, adaptive nature of ET management and identify patients most likely to tolerate and sustain specific ET strategies, as well as those who may benefit from early supportive interventions or alternative regimens.

Several limitations warrant acknowledgment. The modest cohort size and observational design limit statistical power and preclude causal inference. Near-collinearity between initial ET class and menopausal status constrains full separation of drug-class effects from menopausal-status-associated tumor biology; sensitivity analyses supported *WNT7B* and *TP53* most consistently, while the remaining candidates require validation in less correlated cohorts. Effect estimates for gene–ET interactions should be interpreted as exploratory signals warranting validation in larger, independent cohorts.

In summary, our study illustrates how real-world ET trajectories in ER + BC are shaped not only by tumor biology but also by treatment tolerability and patient experience. By integrating molecular profiles with longitudinal ET use and switching, our findings support future frameworks that move beyond static models of endocrine sensitivity toward gene signature strategies balancing efficacy with tolerability, advancing more sustainable approaches to precision endocrine therapy.

## Supplementary Files

This is a list of supplementary files associated with this preprint. Click to download.

- NatureCommunicationsSupplementary.docx

## Figures and Tables

**Table 1:** Baseline clinical and tumor characteristics of patients stratified by initialized endocrine therapy

Characteristics	Total (N = 74; column %)	Initial endocrine therapy (ET) type		P value^a^
		Aromatase Inhibitor (AI)-initiated (n = 49; column %)	Selective estrogen receptor modulator (SERM)-initiated (n = 25: column %)	
**Breast cancer recurrence**				
No	52 (70.27%)	35 (71.43%)	17 (68%)	0.70^b^
Yes	22 (29.73%)	14 (28.57%)	8 (32%)	
**Race and ethnicity**				
White, non-Hispanic	40 (54.05%)	31 (63.27%)	9 (36%)	0.06
Asian, non-Hispanic	17 (22.97%)	8 (16.33%)	9 (36%)	
Hispanic (any race)	17 (22.97%)	10 (20.41%)	7 (28%)	
**Menstrual at diagnosis**				
Premenopausal	27 (36.49%)	8 (16.33%)	19 (76%)	<0.0001
Postmenopausal	47 (63.51%)	41 (83.67%)	6 (24%)	
**Diabetes at diagnosis**				
No	65 (87.84%)	43 (87.76%)	22 (88%)	0.99
Yes	9 (12.16%)	6 (12.24%)	3 (12%)	
**Usage of ovarian suppression therapy**				
No	67 (90.54%)	44 (89.8%)	23 (92%)	0.99
Yes	7 (9.46%)	5 (10.2%)	2 (8%)	
**Histology**				
IDC	55 (74.32%)	34 (69.39%)	21 (84%)	0.26
Non-ductal (ILC/other)	19 (25.68%)	15 (30.61%)	4 (16%)	
**Tumor grade**				
I	4 (5.41%)	2 (4.08%)	2 (8%)	0.17
II	59 (79.73%)	42 (85.71%)	17 (68%)	
III	11 (14.86%)	5 (10.2%)	6 (24%)	
**Cancer stage**				
I/II	54 (72.97%)	35 (71.43%)	19 (76%)	0.79
III	20 (27.03%)	14 (28.57%)	6 (24%)	
**ET switch during follow-up**				
No	44 (59.46%)	30 (61.22%)	14 (56%)	0.80
Yes	30 (40.54%)	19 (38.78%)	11 (44%)	
**Duration of ET (years)**				
< 2	8 (10.81%)	7 (14.29%)	1 (4%)	0.003
2–5	18 (24.32%)	6 (12.24%)	12 (48%)	
5+	48 (64.86%)	36 (73.47%)	12 (48%)	
**Usage of Anastrozole (AI)**				
No	45 (60.81%)	21 (42.86%)	24 (96%)	< 0.0001
Yes	29 (39.19%)	28 (57.14%)	1 (4%)	
**Usage of Exemestane (AI)**				
No	63 (85.14%)	41 (83.67%)	22 (88%)	0.74
Yes	11 (14.86%)	8 (16.33%)	3 (12%)	
**Usage of Letrozole (AI)**				
No	39 (52.7%)	24 (48.98%)	15 (60%)	0.46
Yes	35 (47.3%)	25 (51.02%)	10 (40%)	
**Usage of Tamoxifen (SERM)**				
No	41 (55.41%)	41 (83.67%)	0 (0%)	< 0.0001
Yes	33 (44.59%)	8 (16.33%)	25 (100%)	
**Usage of Toremifene (SERM)**				
No	73 (98.65%)	49 (100%)	24 (96%)	0.34
Yes	1 (1.35%)	0 (0%)	1 (4%)	
**Usage of Fulvestrant (Selective estrogen receptor degrader)**				
No	73 (98.65%)	48 (97.96%)	25 (100%)	0.99
Yes	1 (1.35%)	1 (2.04%)	0 (0%)	
**Age at diagnosis**				
mean (SD)	56.58 (12.75)	62.16 (11.37)	45.64 (6.87)	0.09
**Body mass index at diagnosis**				
mean (SD)	27.71 (5.59)	27.87 (4.96)	27.4 (6.76)	0.39
**Tumor size (cm)**				
mean (SD)	2.89 (1.79)	2.84 (1.92)	2.99 (1.54)	0.53

a P values were derived using Fisher’s exact test for categorical variables and t tests for continuous variables.

b P value was derived using log-rank test.
